# Control of post-translational modifications in antithrombin during murine post-natal development by miR-200a

**DOI:** 10.1186/1423-0127-20-29

**Published:** 2013-05-16

**Authors:** Raúl Teruel, Irene Martínez-Martínez, José A Guerrero, Rocío González-Conejero, María E de la Morena-Barrio, Salam Salloum-Asfar, Ana B Arroyo, Sonia Águila, Nuria García-Barberá, Antonia Miñano, Vicente Vicente, Javier Corral, Constantino Martínez

**Affiliations:** 1 Centro Regional de Hemodonación, University of Murcia, IMIB, Spain, C/Ronda de Garay S/N, 30003, Murcia, Spain

**Keywords:** miRNAs, Sialytransferases, Antithrombin, Post-translational modifications, Microarray, Post-natal development

## Abstract

**Background:**

Developmental haemostatic studies may help identifying new elements involved in the control of key haemostatic proteins like antithrombin, the most relevant endogenous anticoagulant.

**Results:**

In this study, we showed a significant reduction of sialic acid content in neonatal antithrombin compared with adult antithrombin in mice. mRNA levels of *St3gal3* and *St3gal4*, two sialyltransferases potentially involved in antithrombin sialylation, were 85% lower in neonates in comparison with adults. *In silico* analysis of miRNAs overexpressed in neonates revealed that mir-200a might target these sialyltransferases. Moreover, *in vitro* studies in murine primary hepatocytes sustain this potential control.

**Conclusions:**

These data suggest that in addition to the direct protein regulation, microRNAs may also modulate qualitative traits of selected proteins by an indirect control of post-translational processes.

## Background

MicroRNAs (miRNAs) are small non-coding RNAs implicated in the modulation of a large number of physiological and pathological processes [1,2] through a mechanism based on the repression of protein translation or degradation of messenger RNAs [3]. MiRNAs have been recently involved in the modulation of several haemostatic factors such as fibrinogen, tissue factor, and proteins implicated in platelet function [4-6]. Actually, miRNAs can also be involved in the quantitative variations of elements of the haemostatic system observed during development [7]. In humans, levels of haemostatic factors go in constant increase after birth until reaching levels similar to those found in adults within the first year [8-10]. In particular, antithrombin, an anticoagulant serpin crucial in the control of the hemostatic system [11], is significantly reduced (50%) in plasma of neonates in comparison with adults [8]. Despite these differences, neonates maintain a perfect haemostatic equilibrium. Accordingly, a developmental model between neonatal and adult period is ideal to study the mechanisms that regulate haemostatic protein levels and the adaptation of this system to particular conditions. In addition to these quantitative changes, few works have shown that neonatal antithrombin has lower levels of sialic acid than its adult counterpart but the molecular mechanism of this regulation is unknown [12,13]. Thus, the developmental model may also allow to investigating the regulation of *N*-glycosylation by miRNAs and thus to further enlarge the effects of miRNAs in gene regulation.

## Methods

### Mouse samples

Non-inbred Swiss CD1 mice from different litters were sacrificed by cervical dislocation or decapitation at different ages, from day one after birth to adult age. Livers finely dissected were immediately snap-frozen in liquid nitrogen. Blood was anticoagulated with trisodium citrate, centrifuged at 1,500 × *g* for 5 minutes to obtain platelet poor plasma and immediately stored in aliquots at −80°C. All experimental procedures strictly followed the University of Murcia approved Institutional Animal Care guidelines and were approved by the local ethical committee (#C131002043; 15/02/2010).

### Antithrombin levels and activity

Antithrombin activity was determined by chromogenic methods, as previously described [14]. Anti-factor Xa (anti-FXa) assay was performed with pentasaccharide, bovine FXa, and S-2765 chromogenic substrate (Chromogenix, IZASA, Spain). Antithrombin levels were determined by enzyme-linked immunosorbent assay and electro-immunodiffusion (Laurell), as previously reported [15]. Values were expressed as a percentage relative to a pool of citrated plasma from 10 adult control mice (100%).

### Electrophoretic analysis of antithrombin

Mouse plasma samples were run in polyacrylamide gel electrophoresis under denaturing and non-denaturing conditions, blotted onto PVDF membranes, and immunodetected with goat anti-human antithrombin polyclonal antibody (Sigma-Aldrich, Madrid, Spain) and rabbit anti-goat IgG-horseradish peroxidase conjugate (Sigma-Aldrich, Madrid, Spain), with detection via an ECL kit (Amersham Biosciences, Little Chalfont, UK), essentially as described elsewhere [16].

### Isoelectrofocusing

Plasma samples from adult and neonate mice were subjected to isoelectrofocusing (IEF) analysis and electroelution using an OFFGEL fractionator with strips of 12 cm with a pH gradient of 4–7 (Agilent 3100, Agilent Technologies, Madrid, Spain). Each fraction collected was run in SDS-PAGE gel and immunodetected as described above.

### Glycosylation analysis

Plasma from adult and neonate (+1 day) mice (10 μL) were treated with 2 U α2-3,6,8,9 neuraminidase (sialidase) (N 3786, Sigma-Aldrich, Saint Louis, USA) at 37°C for 18 hours in 50 mM sodium phosphate buffer, pH 6.0. Samples were resolved by SDS-PAGE and detected as previously described.

### RNA Isolation

Total RNA was isolated from frozen liver using Trizol® Reagent (Invitrogen, Carlsbad, CA) following manufacturer’s instructions. The RNA concentration and 260/280 ratio were determined by using NanoDrop spectrophotometer (Thermo Scientific, Wilmington, DE) and RNA integrity was verified by lab-on-chip technology using the Experion automated electrophoresis system (Bio-Rad Laboratories, Madrid, Spain).

### MicroRNA microarray

MicroRNAs microarray profiling was performed using total RNA extracted from the liver from one adult mouse (day +50) and one neonate mouse (day +1) using the LC Sciences technology (LC Sciences, Houston, TX). The arrays were designed to detect and quantify miRNA transcripts corresponding to 558 mature miRNAs contained in the Sanger mirBase Release 10.0 (miRMouse 10.0: http://www.mirbase.org/pub/mirbase/10.0/). We used two chips (1 and 2) in which RNAs from each sample were labeled either with cy3 or with cy5. The signal values were derived by background subtraction and normalization. Additional details on the array are available elsewhere [7].

### In silico studies

Several web databases and algorithms of miRNA target prediction were used for the search of miRNA targeting sialyltransferases. We essentially used TargetScan [17] (release 5.1: http://www.targetscan.org), which provides the prediction results computed by the TargetScanS algorithm, PicTar ( http://www.pictar.mdc-berlin.de) [18], and miRanda ( http://www.microrna.org/microrna/home.do) [19].

### Murine hepatocyte primary culture

Hepatocytes were isolated from livers of Swiss CD1 mice using a modified version protocol from Wu et al. [20]. Mice were anesthetized with an intraperitoneal injection of a ketamine/xylazine mixture. A 24G clear cannula was inserted into the posterior vena cava and secured with a ligature. A second ligature was placed around the anterior vena cava, between the liver and the heart, and the portal vein was severed, allowing outflow of solution. The liver was then perfused at 37°C with oxygenized HBSS (in mM: 137 NaCl, 5.4 KCl, 0.8 MgSO4.7H2O, 0.3 NaHPO4.2H2O, 0.44 KH2PO4, 26 NaHCO3, pH 7.4) 3 min at 5 mL/min and 5 min at 7 mL/min. The perfusion solution was then changed to HBSS supplemented with 4 mM CaCl2 and containing 0.12% collagenase (Sigma-Aldrich, Madrid, Spain) for 8 min at 5 mL/min. The liver was additionally incubated with HBSS with 0.12% collagenase for 15 min, filtered through a cell strainer (100 μm from Becton Dickinson, Madrid, Spain) and hepatocytes were isolated by repeated 50 × *g* centrifugations. Viability was assessed using trypan blue to be >90% in all the cases. Six-well plates were pre-coated with 50 μg/mL collagen from Stemcell (Grenoble, France) for 12 h at 4°C and cells were seeded at 250,000/well.

### Hepatocyte transfection

Primary hepatocytes were maintained in DMEM/F12 supplemented with 10% fetal bovine serum at 37°C in a humidified incubator with 5% CO2. Cells were pre-cultured for 24 h in complete medium without antibiotics and transfected at 40-60% confluence with 100 nmol/L of precursor molecules for miR-17-3p, miR-200a, and negative scrambled control (Applied Biosystems, Madrid, Spain) by using siPORT™ NeoFX™ transfection agent (Applied Biosystems, Madrid, Spain). The cells were collected 48 hours after transfection and total RNA was extracted.

### qRT-PCR and validation assays

Total RNA from mouse livers and from transfected hepatocytes was isolated using Trizol® Reagent (Invitrogen, Madrid, Spain). RNA integrity was verified using bioanalyzer (Bio-Rad, Madrid, Spain). RNA samples were stored at −80°C until used in the experiments. The miRNA and mRNA quantification were carried out as previously described [5]. For *St3gal3*, *St3gal4*, and *St6gal1,* as well as for *serpinc1* transcripts relative quantification, retrotranscription reactions were performed using 100 ng of total RNA for each sample according to the manufacturer instructions (SuperScript First Strand, Invitrogen, Madrid, Spain). One set of primers and a probe were chosen from the Applied Biosystems list of TaqMan® Gene Expression Assays for these sialyltransferases (Hs00544033_m1, Hs00920871_m1, and Hs00949382_m1, respectively). For *serpinc1* expression was measured using assay Hs00166654_m1 (Applied Biosystems). Sialyltransferase mRNA expression analysis was performed in triplicate for each sample. Expression of β-actin (Hs99999903_m1) was used as endogenous reference control. The PCR reactions were performed using an LC480 Real Time PCR system (Roche Applied Science, Barcelona, Spain). We employed the 2^-ΔCt^ method to calculate the relative abundance of miRNA and mRNA compared with endogenous control expression. Ct is the Threshold Cycle and ΔCt = Ct sample gene - Ct endogenous control.

MiRNA assay kits for miR-200a (Applied Biosystems, Madrid, Spain) were used to validate expression levels in mouse hepatocytes during post-natal development (neonates day+1, n=14; adults day+50, n=5). Expression of U6 snRNA (Applied Biosystems, Madrid, Spain) was used as endogenous reference control.

## Results

### Quantitative differences of antithrombin between neonate and adult mice

**Figure 1:**
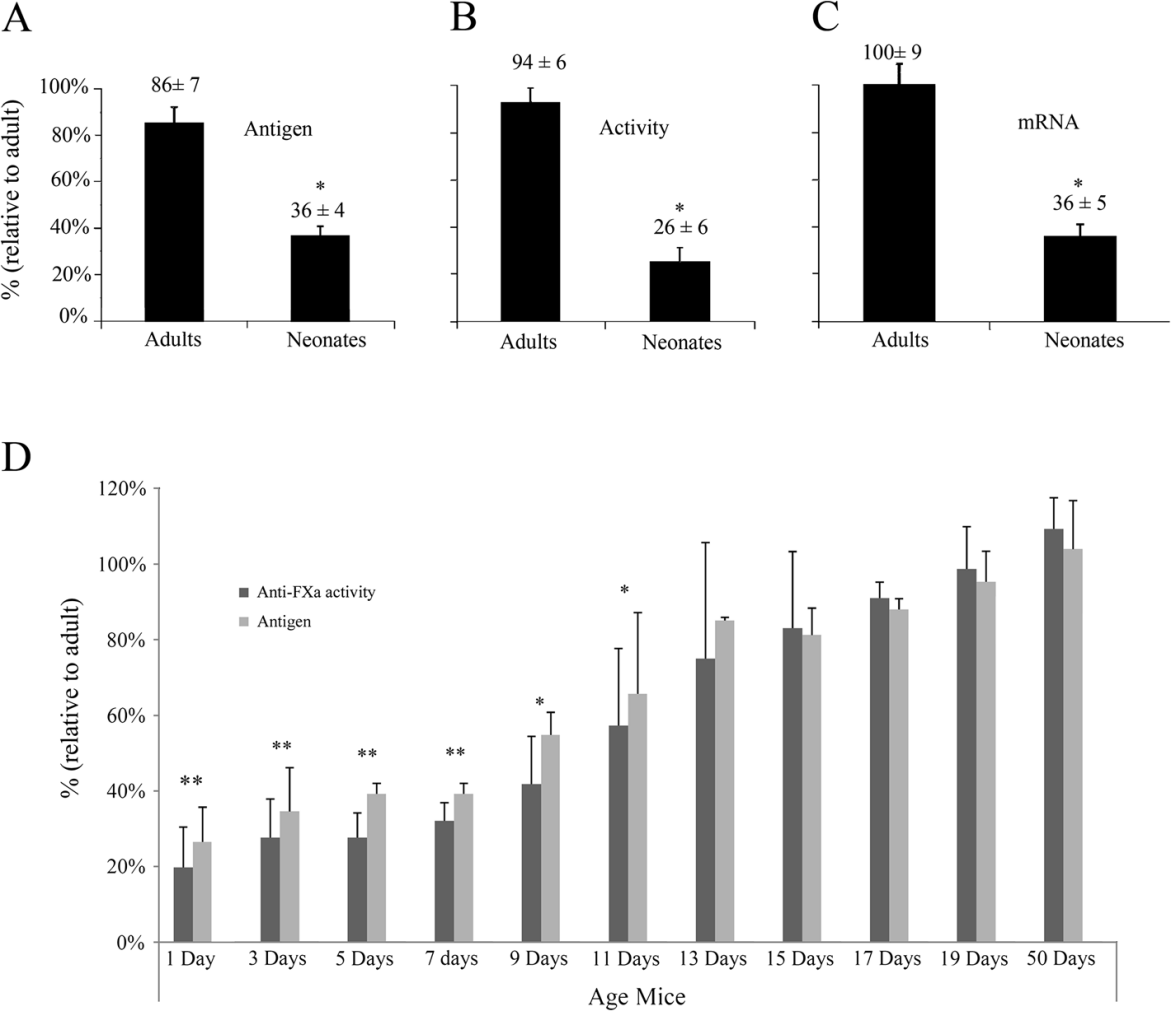
**Expression of antithrombin in neonate and adult mice.** Levels of antithrombin in neonate (day +1, n=13) relative to adult (day +50, n=6) mice: (**A**) plasma antigen, (**B**) plasma anti-FXa activity, and (**C**) *serpinc1* mRNA. (**D**) Levels of plasma antithrombin antigen and plasma anti-factor Xa activity at different stages of development relative to adult stage (n=5 for each age). Differences were analyzed by means of Student's *t* test taking the adult group as a reference group. Statistical significance was taken as p<0.05. **Both measurements were statistically significant. *Anti-FXa activity was statistically significant.

Antithrombin levels in plasma of neonates (day+1) were 60% lower than in adults (day+50) [36±4% (n=13) *vs.* 86±7% (n=6)] (Figure 1A). As expected, correlating values were observed in antithrombin activity [neonate (n=13): 26±6% *vs.* adult (n=6): 94±6%] (Figure 1B). We checked the association between these values and *serpinc1* mRNA levels in liver. As shown in Figure 1C, *serpinc1* mRNA levels in neonates and adults [36±5% (n=13) *vs.* 100±9% (n=6)] matched with previously published data [7], and correlated with antigen and functional levels in plasma. In order to better delineate the variations observed along the development, we determined the antigenic levels and activity of antithrombin of three different mice litters from day one after birth to adult age. Our results showed that antithrombin antigen and activity levels paralleled. At day+13 after birth, antithrombin levels were similar to those observed in adults (Figure 1D).

### Qualitative differences of antithrombin in neonate and adult mice

**Figure 2:**
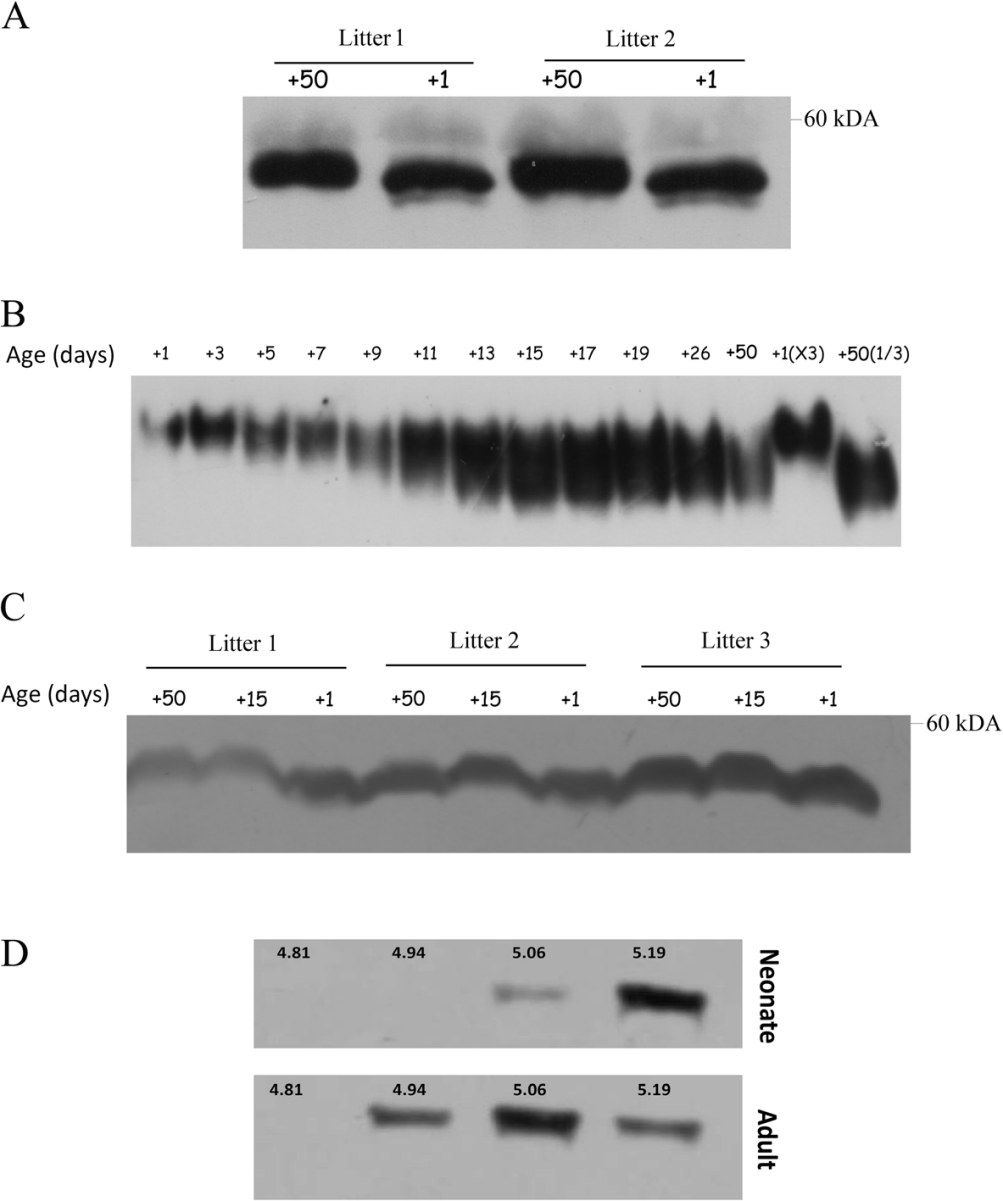
**Electrophoretic features of plasma antithrombin in neonate and adult mice during post-natal development.** Plasma (1 μL) from mice at different stages of development (the number indicates the age in days) were evaluated in PAGE gels under SDS denaturing (**A**, **C**) or native (**B**) conditions. In order to check for potential biases under native conditions due to antithrombin loading charge, the two last lanes contained 3 μL (3x) of neonate (day +1) plasma and 0.3 μL (1/3) of adult (day +50) plasma. (**D**) SDS-PAGE of selected fractions obtained after isoelectrofocusing and electroelution of plasma antithrombin from neonate and adult mice [Isoelectric point (pI) values are indicated]. Images are representative of different experiments (**A**, **B**, and **C**: n=3; **D**: n=2).

SDS-PAGE analysis of plasma antithrombin revealed that plasma antithrombin from neonate mice had a lower molecular weight than its adult counterpart (Figure 2A). We next performed native gel electrophoresis with plasma samples extracted at different times during mouse post-natal development. Newborn mice had a plasma antithrombin with slower migration than the adult one (Figure 2B), and this result is compatible with a lower global negative charge in neonate’s antithrombin. Plasma antithrombin concentration was not responsible for the differences in electrophoretic mobility (Figure 2B, last lanes). Interestingly, we observed that at day+15, plasma antithrombin from neonate had the same electrophoretic characteristics (Figure 2B) and size (Figure 2C) than adult antithrombin.

To further evaluate the differences of neonatal and adult antithrombins, we performed IEF of plasma from neonate and adult mice. Our results showed that neonates expressed more antithrombin isoforms with higher pI (5.19) and lacked of isoforms with lower pI (4.94) (Figure 2D). These results were in accord with those obtained in native electrophoresis.

### Antithrombin glycosylation

**Figure 3:**
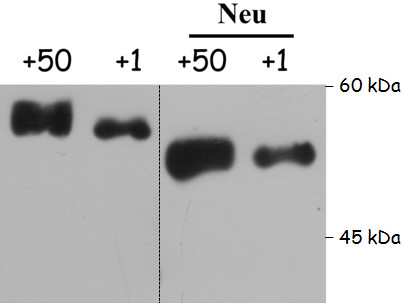
**Glycosylation of antithrombin from neonate (day +1) and adult (day +50) mice.** The image, representative of two different experiments, shows the electrophoretic pattern of plasma antithrombin treated or not with neuraminidase (Neu).

In order to evaluate the role of glycosylation in the qualitative changes of neonate’s antithrombin, we treated plasma from adult and neonate mice with neuraminidase. This treatment rendered the same electrophoretic mobility in SDS-PAGE gels for adult’s and neonate’s antithrombins and sustained an incomplete content of sialic acid for neonate’s antithrombin (Figure 3) that may explain the different migration of neonate’s antithrombin observed in SDS, native electrophoresis, as well as the IEF results (Figure 2).

### Expression of sialyltransferases potentially involved in glycosylation of antithrombin

**Figure 4:**
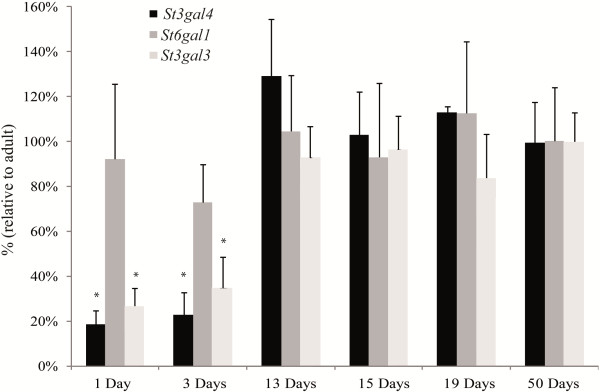
**Levels of selected sialyltransferases mRNA and of miR-200a in neonate and adult liver.** Levels of mRNA from three sialyltransferases (*St3gal3 and St3gal4*, and *St6gal1*) at different stages of development relative to adult stage (n=5 for each age) were measured by qRT-PCR and normalized with respect to β-actin mRNA.

There are three different sialyltransferases able to sialylate *N*-linked glycoproteins like antithrombin, *i.e.* St3gal-III, St3gal-IV, and St6gal-I. In order to evaluate if any of these three sialyltransferases were down regulated in neonates, we measured their mRNA levels in liver from neonates and adults by qRT-PCR. As shown in Figure 4, our results indicated an ~85% reduction in neonates in comparison with adults for *St3gal3* and *St3gal4* expression, whereas levels of *St6gal1* mRNA remained unchanged. As it happened for antithrombin, mRNA levels of *St3gal3* and *St3gal4* were similar to those observed in adults at day+13 after birth (Figure 4).

### Regulation of St3gal3 and St3gal4 by miR-200a

**Figure 5:**
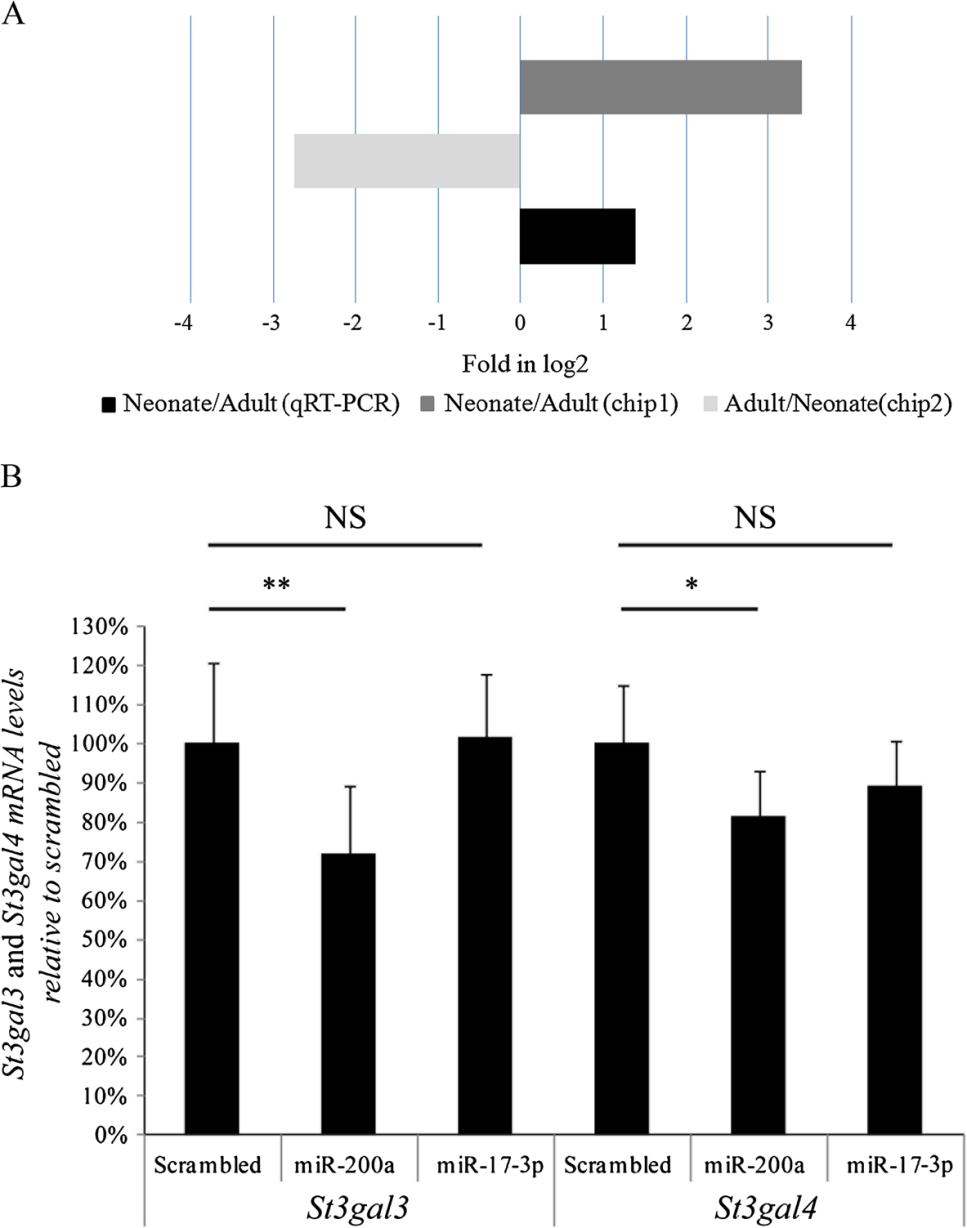
**Role of miR-200a in the control of *St3gal3 and St3gal4 *in murine primary hepatocytes.** (**A**) Fold increase of miR-200a in neonate *vs*. adult liver was determined in a miRNA array (chip 1 and chip 2) and validated by qRT-PCR in livers from 5 adult and 14 neonate mice. (**B**) Primary hepatocytes from adult mice were transfected with precursor molecules for miR-200a and miR-17-3p and with a scrambled oligonucleotide at 100 nmol/L. After 48 h, total RNA was purified and levels of *St3gal3 and St3gal4* were measured by qRT-PCR and normalized with respect to β-actin mRNA. The normalized data were expressed as changes relative to the data of the cells transfected with scrambled pre-miR and set as 100%. Differences were analyzed by means of Student's *t* test. Statistical significance was taken as *p<0.05, **p<0.01, NS: not statistically significant. The data shown are expressed as mean ± SD, representative of four independent experiments with three replicates each.

One hypothesis to explain the variation of the levels of *St3gal3* and *St3gal4* during post-natal development may reside in a miRNA-dependent regulation. Using target predicting algorithms, we found that miR-200a may target *St3gal3* and *St3gal4* (Table 1) and thus it was a valuable candidate to explain the lower sialylation of antithrombin in neonate mice. Interestingly, the analysis of the subtractive miRNA array revealed that miR-200a is over-expressed in neonates in comparison with adults in both chips. Moreover, validation studies in 5 adults and 14 neonates by qRT-PCR confirmed this result (Figure 5A). The next step to demonstrate the potential regulation of *St3gal3* and *St3gal4* by miR-200a was to perform transfection studies of primary hepatocytes from adult mouse with miR-200a. Interestingly, this procedure provoked a significant reduction of *St3gal3* and *St3gal4* (31% and 20%, respectively), whereas no effect was observed when a scrambled oligonucleotide was employed or when cells were transfected with miR-17-3p, a miRNA expressed at high levels in neonates that, according to *in silico* predictions, does not modulate these sialyltransferases (Figure 5B).

**Table 1:** **miR-200a putative target site in *St3gal3 *and *St3gal4 *mRNA using different target prediction software**

**miRNA target prediction software**	**Parameters**	***St3gal3***	***St3gal4***
TargetScan (release 5.2) [17]	Seed match	7mer-m8	7mer-m8
	Context score percentile	85	75
	P_CT_	0.35	0.17
Pictar [18]	Score	4.67	-
	Target site number	1	-
	Free energy (Kcal/mol)	−21.4	-
microRNA.org [19]	mirSVR score	−0.9262	−0.2421
	PhastCons score	0.5877	0.5271

## Discussion

Antithrombin is the main endogenous anticoagulant and, thus, its role in regulating haemostasis is absolutely essential. Indeed, complete antithrombin deficiency is incompatible with life and partial deficiency is an important risk factor for developing venous thrombosis [21]. Besides its role in haemostasis, antithrombin may also regulate other important physiological processes such as inflammation, angiogenesis or apoptosis [22-24]. Intriguingly, the levels of antithrombin in neonates are severely reduced in comparison with adults without relevant physiological consequences [8,9,25]. This study confirms that the difference not only relies on protein expression levels but also in post-translational modifications. Here, we studied the expression, features, and functionality of antithrombin in a mouse model to deepen into the impact of post-translational modifications of this protein in developmental haemostasis.

Our previous results suggested that the lower levels of antithrombin in neonate mice are mainly explained by a concomitant reduction of mRNA in hepatocytes [7]. In addition, electrophoretic data in the present study suggest that the lower molecular weight of antithrombin from neonates is due to a post-translational modification: an abnormal *N*-glycosylation (Figures 2 and 3). Our results show that the sialic acid content of antithrombin is smaller in neonates than adults. Unfortunately, we were unable to perform fine glycomic studies to calculate the exact sialic acid content of neonate antithrombin due to the large amount of purified protein that is required for this procedure. Interestingly, a reduced sialylation of antithrombin has also been described for antithrombin in chicken and sheep neonates [12,13]. These data strongly suggest that this has to be a process highly regulated in different species. Aiming to identify the mechanisms involved in such control, we evaluated the mRNA levels of three sialyltransferases potentially involved: *St3gal3*, *St3gal4*, and *St6gal1*. Indeed, St6gal-I performs α2-6 sialic acid linkage as that present in antithrombin [26]. Accordingly, this enzyme seems to be the main responsible for the sialylation of antithrombin. However, in St6gal-I KO mice, St3gal-IV, that performs α2-3 linkages, may also achieve α2-6 linkages in von Willebrand factor [27]. In addition, a study by Fan et al. revealed that recombinant human antithrombin expressed in baby hamster kidney cells is fully sialylated containing α2-3 linkage [28]. Thus, it is worth suggesting that the lower levels of sialic acid in neonate’s antithrombin might be explained by the reduced expression of *St3gal3* and *St3gal4*. Further experiments are necessary to clarify these issues.

The next step to understand the mechanism responsible for these differences was the identification of the element(s) controlling the levels of these sialyltransferases. In this framework, the recent report suggesting that some conserved genes implicated in glycosylation pathway may be regulated by miRNAs during animal development [29], reveals miRNAs as potential candidates. n fact, *in silico* searching identified miR-200a as an excellent regulator of *St3gal3* and *St3gal4*. Interestingly, the levels of this miRNA during development show a fully compatible change (overexpressed in neonate mice, but reduced expression in adults). The final proof indicating the control of these sialyltransferases by miR-200a was obtained by transfecting this miRNA in adult primary hepatocytes. These experiments suggest that miR-200a may be in part implicated in the regulation of *St3gal3* and, in a lesser degree, in the regulation of *St3gal4*, as predicted by *in silico* studies (Table 1). Specificity of this regulation is further suggested by the lack of effect of another miRNA overexpressed in neonate’s liver, miR-17-3p. However, other mechanisms and additional miRNAs still to characterize may be involved in the reduced expression of these two sialyltransferases in neonate mice.

Finally, it would be of great interest to evaluate whether or not these qualitative modifications regulated indirectly by miRNAs could have functional significance apart of contributing to an increased clearance [30]. In our case, it is necessary to investigate the functional relevance of the lower sialylation in antithrombin, not only on the anticoagulant function, which might contribute to explain the dramatic change of the haemostatic system after birth, but also on other functions of this molecule.

## Conclusions

Our results supported by those of Kahai et al. showing that UDP-N-acetyl-alpha-D-galactosamine:polypeptide N-acetylgalactosaminyltransferase 7 (GalNAc-T7) is inhibited by miR-378 with consequences in the rate of osteoblast differentiation [31], open new and interesting perspectives, as the regulation of proteins involved in *N*-glycosylation (and potentially any other post-translational modification) of antithrombin (and extensively other proteins) may be done by miRNAs. The role of miRNAs in diseases and physiological processes is therefore not restricted to the direct control of proteins of one system (in this case, the haemostatic system), but could be extended to an indirect effect by affecting elements involved in transcriptional [32,33], translational or post-translational processes.

## Competing interests

The authors declare that they have no competing interests.

## Authors’ contributions

RT, IMM, MEMB, SS-A, and SA performed biochemical assays (WB, IEF; qRT-PCR). JAG and NGB performed work with mice. ABA and RGC performed i*n vitro* assays. AM measured protein levels and activities. RT, JC, and CM designed the research, analyzed the results, and wrote the paper. VV critically read the manuscript. All authors read and approved the final manuscript.
